# Editorial: Web-based and mobile-based interventions for perinatal mental health

**DOI:** 10.3389/fgwh.2023.1207447

**Published:** 2023-05-17

**Authors:** Ana Fonseca, Emma Motrico

**Affiliations:** ^1^ University of Coimbra, Center for Research in Neuropsychology and Cognitive-Behavior Interventions, Coimbra, Portugal; ^2^ Department of Psychology, Loyola Andalusia University, Seville, Spain

**Keywords:** perinatal mental health, web-based intervention, mobile-based intervention, eHealth, mental health

**Editorial on the Research Topic** Web-based and mobile-based interventions for perinatal mental health

Perinatal mental disorders (e.g., depression and anxiety) are the most frequent complications of pregnancy and postpartum, affecting an estimated one in 10 women in high-income countries and one in five in low- and middle-income countries (1–3). With the COVID-19 pandemic, there was an increase in the prevalence of depression and anxiety symptoms in perinatal women compared to perinatal women prior to the pandemic, due to increased stress, financial stress and low support, among other aspects (4). Perinatal mental health disorders are associated with adverse outcomes for both the mother and the child (5), which translates into higher economic and societal burden (6).

The public health impact of perinatal mental health disorders has been increasingly recognized (7), highlighting the need to focus on the development and implementation of effective prevention and treatment programs targeting maternal mental health. However, the help-seeking rates for perinatal mental health problems are very low (8), due to practical (e.g., financial or job restrictions, lack of assistance with childcare) and attitudinal barriers (e.g., stigma) (9), but also for lack of specialized mental health services, particularly in low and middle-income countries (10).

In recent years, web-based and mobile-based interventions have created new opportunities for improving mental health, offering many advantages over traditional face-to-face interventions through reduced cost, increased accessibility, convenience, and privacy (11). In the perinatal context, web-based and mobile-based interventions may be an effective way to improve women's accessibility and use of mental healthcare (12), and these interventions seem to be acceptable and useful to women (13).

This Research Topic includes one scoping review and three original studies targeting web-based and mobile-based interventions for postpartum depression prevention or treatment.

The scoping review conducted by Syed et al. synthesizes existing knowledge about maternal and perinatal health in low- and middle-income countries to investigate how country strategies evolved to improve maternal survival and wellbeing, discussing strategic directions for the future.

In the first original study, Xavier et al. examined the patterns of usage of the *Be a Mom program*, a self-guided web-based intervention targeting both women presenting high and low-risk for postpartum depression. The results of the study suggested that only about one-third of the participants completed the full program, although around 20% were partial completers. Despite this, women rate the *Be a Mom* program as acceptable and useful. The high dropout rates of self-guided web-based programs were already found in prior studies of web-based interventions targeting postpartum depression and are an important challenge to the implementation of such interventions. The implications of these findings were discussed.

In the second original study, Barrera et al. presented the results of the *Mothers and Babies Online Course,* a digital adaptation of the original Mothers and Babies Course. The paper focused on the user's feedback about the program, both considering women in the perinatal period and health providers who want to provide support to their patient communities. The results highlighted a high number of non-perinatal users enrolled, which suggests that health providers may also seek perinatal mental health resources. Moreover, the study also explored users' engagement in the program, and reflected on how the current data is a crucial step in intervention's development prior to the test of efficacy of digital tools.

In the third original study, Tang et al. described the results of the users' experience of a mobile-based intervention for postpartum depression treatment, the *MamaLift Plus*. The mobile-based intervention was found to be an acceptable, usable, and feasible intervention to address symptoms of postpartum depression. The users' perceptions were also important to inform further developments of the mobile application, especially concerning users' engagement.

In summary, the original studies published in this Research Topic use different approaches to assess aspects of use and acceptability of web-based/mobile-based interventions targeting perinatal depression, highlighting the importance of considering the users' perspective in the different stages of development and evaluation of digital tools for mental health prevention and treatment.
